# A novel marker of persistent left ventricular systolic dysfunction in patients with peripartum cardiomyopathy: monocyte count- to- HDL cholesterol ratio

**DOI:** 10.1186/s12872-019-1100-9

**Published:** 2019-05-15

**Authors:** Firdevs Aysenur Ekizler, Serkan Cay

**Affiliations:** 1 Department of Cardiology, Turkiye Yuksek Ihtisas Training and Research Hospital, Ankara, Turkey; 2 Türkiye Yüksek İhtisas Hastanesi Kardiyoloji Klinigi, Ankara, 06100 Turkey

**Keywords:** Left ventricular recovery, Marker, Monocyte-to-HDL cholesterol ratio, Peripartum cardiomyopathy

## Abstract

**Background:**

Peripartum cardiomyopathy (PPCM) is a rare but potentially life-threatening complication of pregnancy. There is limited data regarding the predictors of persistent left ventricular (LV) systolic dysfunction. Recently, monocyte-to-high density lipoprotein (HDL) cholesterol ratio (MHR) has emerged as a novel indicator of inflammation and oxidative stress. We aimed to assess the predictive value of MHR on LV recovery in patients with PPCM.

**Methods:**

A total of 64 patients with PPCM who admitted to our tertiary reference hospital between 2009 and 2017 were retrospectively analyzed in this study. Demographic and clinical data, laboratory parameters and echocardiographic findings were recorded. The duration of follow-up was at least 12 months after diagnosis for all participants. Recovery of LV systolic function was defined as the presence of LV ejection fraction (LV EF) > 45%. Univariate analysis was used to determine the significant predictors of persistent LV systolic dysfunction (non-recovery). A receiver operating characteristic (ROC) curve was used to establish the cut-off values for predictors.

**Results:**

The mean follow-up duration was 72.1 ± 5.5 months. Of the 64 patients, 35 (55%) had persistent LVSD at their last follow-up while 29 (45%) showed LV EF improvement. The baseline MHR levels were significantly higher in the non-recovery group (*P* < 0.001). In univariate analysis, increased MHR levels (odds ratio:1.17; 95% confidence interval, 1.01–1.35; *P* < 0.001) significantly predicted LV non-recovery. Using a cut-off level of 9.73, MHR predicted persistent LV systolic dysfunction with a sensitivity of 89% and specificity of 79%. Besides, lower baseline LVEF increased WBC and CRP levels were identified as predictors of LV non-recovery.

**Conclusions:**

Our data firstly indicated that elevated MHR was a significant predictor of persistent LV systolic dysfunction in PPCM. The MHR might contribute to determining high-risk patients with PPCM.

## Background

Peripartum cardiomyopathy (PPCM) is an uncommon but potentially life-threatening complication of pregnancy [1]. It is defined as, an occurrence of unexplained heart failure with reduced EF, usually < 45%, presenting toward the end of the pregnancy or in the first months postpartum in previously healthy women, where no other cause of heart failure is found [2]. PPCM is endemic in parts of Africa, but the actual incidence is unknown [3]. The exact pathophysiological mechanism that leads to PPCM is an unknown but genetic basis, viral myocarditis, abnormal immune or hemodynamic response to pregnancy, nutrient deficiency, increased oxidative stress and inflammation have all been proposed [2, 4–7]. The clinical course is markedly heterogeneous. PPCM might lead to progressive heart failure, thromboembolic complications, malignant arrhythmias, and even death [8]. On the other side of the spectrum, PPCM is associated with high likelihood of LV recovery (up to 41% depending on the race, study size and follow-up period) [1, 9, 10]. The differences in the clinical course have induced clinical researchers to identify baseline predictors of outcome. Predictors of persistent left ventricular systolic dysfunction (LVSD) are inconsistently defined and include lower baseline LV ejection fraction (LV EF), late diagnosis, older age, black race and elevated plasma markers of inflammation [1, 2, 9]. Therefore, the ability to identify early predictors of prognosis in patients diagnosed with PPCM is very important in risk stratification, preventing complications and improving outcomes.

Considering the possible role of oxidative stress and inflammation in the initiation and progression of PPCM, various inflammatory biomarkers including C-reactive protein (CRP), TNF-alpha and interleukin-6 have been studied and demonstrated to be associated with this unique form of heart failure. Recently, monocyte-to-high density lipoprotein (HDL) cholesterol ratio (MHR) has emerged as a novel and widely available inflammation and oxidative stress-based marker. In several studies, MHR has been reported as a significant prognostic marker in various cardiovascular diseases [5, 6, 8]. However, the prognostic value of MHR in patients with PPCM has not yet been described. Thus, in the current study, we sought to investigate the predictive value of baseline MHR on patients with PPCM.

## Methods

### Study population

A total of 64 consecutive patients diagnosed with PPCM in our tertiary reference center between April 2009 and May 2017 were included in this retrospective analysis. PPCM was defined as an occurrence of unexplained heart failure with LVEF < 45%, presenting toward the end of pregnancy or in the first months after delivery in previously healthy women [1]. All women were at least 18 years of age. Exclusion criteria were having a history of cardiomyopathy, severe organic valvular heart disease, significant coronary heart disease (≥50% luminal diameter stenosis in at least 1 major coronary arteries and their branches), clinical conditions other than cardiomyopathy that could increase plasma levels of inflammatory markers such as active cancer, active infection, chronic inflammatory disease, chronic antihyperlipidemic treatment, and patients without a recorded measurement of admission laboratory parameters were excluded from this study. Data regarding clinical and demographic features and laboratory parameters were obtained from the patients’ medical records. The follow-up duration was at least 12 months after diagnosis of PPCM for all patients. Standard, 2-dimensional and Doppler echocardiographic measurements were performed in all women at the time of diagnosis and the last follow-up visit. LVEF was measured using the Modified Simpson rule. Recovery of LV systolic function was accepted as the presence of LVEF >45%, whereas non-recovery (persistent left ventricular systolic dysfunction) was defined as the presence of LVEF≤45% at last follow-up visit.

Fasting venous blood samples were collected at baseline in pre-cooled EDTA tubes for the hematological test and dry tubes for biochemical analyses. The HDL-C concentration was determined by selective solubilization method (Determiner L HDL, Kyowa Medex, Tokyo, Japan). WBC counts were measured using an automated hematology analyzer XE-1200 (Sysmex, Kobe, Japan). Baseline MHR was calculated by dividing the absolute count of the monocytes by the complete counts of the HDL-C. The local ethics committee approved the study protocol.

### Statistical analysis

Data were analyzed using the SPSS 20.0 Statistical Package Program for Windows (SPSS, Inc., IL, USA). Continuous variables were reported as mean ± SD and median with interquartile ranges as appropriate and categorical variables were expressed as the number of patients and percentages. The Shapiro-Wilk test was used to test the normality of distribution. The comparisons between groups were evaluated by using Student’s t-test for normally distributed variables and Mann-Whitney U test for variables without normal distribution. The Chi-square or Fisher’s Exact test was used to compare categorical variables as appropriate. A univariate Logistic regression analysis was used to assess the capability of the individual variables to predict persistent LVSD. We did not use multivariate analysis as the small sample size may limit the power of the statistical test in revealing independent predictors. The ROC curve analysis was used to establish an optimum cut-off level of admission MHR values to predict persistent LVSD. A *p*-value < 0.05 (using a two-sided test) was considered significant.

## Results

Sixty-four patients were identified with the diagnosis of PPCM. The mean age at diagnosis was 29.2 ± 6.0 years. For the entire study population, 15.6% had a history of chronic or gestational hypertension, 10.9% had a family history of dilated cardiomyopathy, 4.7% were diabetic, 17.2% were dyslipidemic, and 3.1% had a history of CAD. The percentage of women in each New York Heart Association functional class (I to IV) at admission was 8.6, 62.1, 25.9, 3.7%, respectively. The mean follow-up duration was 72.1 ± 5.5 months. The majority of the women were treated with optimal therapy for heart failure (beta blockers and angiotensin-converting enzyme inhibitors (ACEI)/angiotensin receptor blockers (ARBs)). There was no difference in the use of beta-blockers and ACEI/ ARBs in patients with and without recovery of LV function. None of the PPCM patients received bromocriptine.

**Fig. 1:**
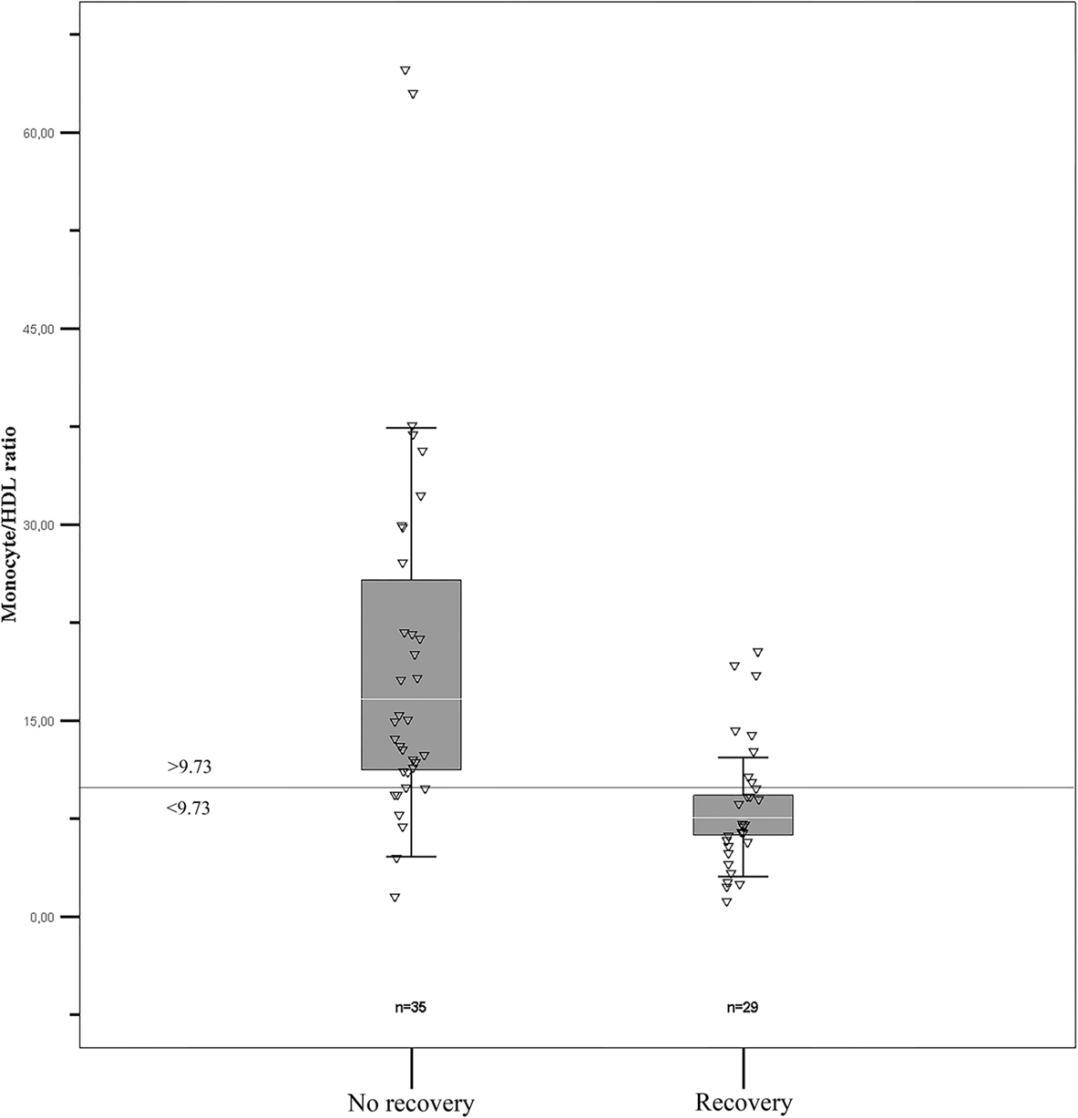
Monocyte to-HDL-cholesterol ratio according to left ventricular recovery**.** Monocyte to -HDL-cholesterol ratio was significantly higher in nonrecovery group**.** HDL, high density cholesterol

There were 29 (45.3%) women who had LV recovery (recovery group), while 35 (54.7%) women had persistent LV systolic dysfunction (non-recovery group) at their last follow-up. Baseline clinical and laboratory characteristics of patients with and without a recovery in LVEF are shown in Table 1. Five women among the recovery group received an intracardiac defibrillator (ICD) early in their follow-up (*n* = 5/29, 17%), while 15 (*n* = 15/35, 43%) devices were present in the non-recovery group. ICD was implanted for primary prevention in 19 women and secondary prevention in one woman. There were appropriate ICD shocks for VT in four (11.4%) patients with persistent LVSD and one (3.4%) patient with LV recovery. In the non-recovery group, there were five deaths during follow-up (four from progressive LV systolic dysfunction and one from stroke). During the follow-up period; one left ventricular assist device implantation, one heart transplantation and six embolic events (four among non-recovery group and two among recovery group) occurred in patients. There was no significant difference between recovery and non-recovery groups concerning to age and co-morbidities such as; hypertension, dyslipidemia, diabetes mellitus, chronic obstructive pulmonary disease (COPD), coronary artery disease (CAD). Besides laboratory, parameters were similar between the two groups (Table 1). However, the LV non-recovery group had significantly higher plasma monocyte level, WBC count, HDL-C, CRP, and MHR. The comparison between recovery and non-recovery group according to admission MHR values is shown in Fig. 1. Patients with more severe LV dysfunction at study entry had significantly lower LVEFs at the last follow-up visit.

**Table 1:** Baseline clinical and laboratory characteristics of patients with and without recovery in LVEF

Characteristic	Nonrecovery Group	Recovery Group	*P* value
	(*n* = 35)	(*n* = 29)	
Age at diagnosis (years) (SD)	29.8 ± 6,0	28.5 ± 6,0	0.417
Hypertension, n (%)	5 (14.3)	5 (17.2)	0.746
Dyslipidemia, n (%)	8 (23.5)	3 (10.3)	0.169
CAD, n (%)	0 (0)	2 (7.1)	0.124
Diabetes, n (%)	3 (9.1)	0 (0)	0.102
COPD, n (%)	2 (6.3)	0 (0)	0.178
Family history, (%)	6 (18.8)	1 (3.6)	0.109
ICD, n (%)	15 (42.8)	5 (17.2)	**0.043**
True ICD therapy, n (%)	4 (11.4)	1 (3.4)	0.236
ACEI / ARB, n (%)	29 (82.9)	24 (82.8)	0.992
B-blockers, n (%)	28 (80)	23 (79.3)	0.946
Digoxin, n (%)	11 (31.4)	7 (24.1)	0.518
Heart rate (bpm)	82.3 ± 14.1	81.4 ± 13.9	0.793
Systolic blood pressure (mmHg)	120.1 ± 13.4	121.7 ± 11.9	0.610
Diastolic blood pressure (mmHg)	77.4 ± 5.9	77.6 ± 5.6	0.843
Body- mass index (kg/m^2^)	24.0 ± 4.7	25.0 ± 4.7	0.377
Baseline LV EF ^a^ (%)	29.0(21.0–35.0)	36.0(33.5–39.5)	**< 0.001**
Uric acid ^a^ (mg/dl)	6.8(4.9–8.8)	5.6 (4.8–6.8)	0.076
Urea ^a^ (mg/dl)	30.0(20.0–38.0)	23.5(19.0–27.7)	0.053
Creatinine ^a^ (mg/dl)	0.74(0.63–0.94)	0.71(0.59–0.82)	0.131
GFR(SD) (mL/m^2^)	94.0 ± 35.5	102.1 ± 29.9	0.455
Total cholesterol ^a^ (mg/dl)	171(132–212)	159(144–184)	0.278
Triglyceride ^a^ (mg/dl)	136.0(88.0–180.0)	97.0(84.0–128.0)	**0.049**
LDL-C ^a^ (mg/dl)	102.0(74.0–13.0)	85.0(73.5–112.5)	0.200
HDL-C (mg/dl) (SD)	39.9 ± 14.6	52.0 ± 13.2	**0.001**
Albumin (mg/dl) (SD)	4.0 ± 0.6	4.1 ± 0.6	0.390
CRP ^a^ (mg/dl)	6.2(2.4–19)	2.1(0.9–3.3)	**< 0.001**
Hemoglobin (g/dl) (SD)	12.5 ± 1.7	12.8 ± 1.9	0.53
WBC ^a^ (× 10^3^ μL)	8.3(7.2–10.5)	7.5(5.8–8.5)	**0.021**
Neutrophil ^a^ (×10^3^ μL)	5.0(4.1–6.6)	4.8(3.7–5.3)	0.113
Lymphocyte ^a^ (× 10^3^ μL)	2.4(1.8–2.9)	2.0(1.4–2.6)	0.084
Monocyte (×10^3^ μL) (SD)	0.63 ± 0.18	0.47 ± 0.14	**< 0.001**
Monocyte / HDL ratio ^a^	15.5(10.8–28.5)	8.8(6.4–13.8)	**< 0.001**
MHR > 9.73, n (%)	31 (88.6)	11 (37.9)	**< 0.001**

Bold data displays statisticially significant difference (*p* < 0.05)

*ACEI* angiotensin-convertingenzyme inhibitör*, ARB* angiotensin receptor blocker*, CAD* coronary artery disease, *COPD* chronic obstructive pulmonary disease, *CRP* C-reactive protein, *GFR* glomeruler filtration rate, *HDL-C* high-density lipoprotein cholesterol*, ICD* intracardiac defibrillator, *LDL-C* low-density lipoprotein cholesterol, *LVEF* left ventricular ejection fration,*MHR* monocyte to HDL cholesterol ratio, *SD* standart deviation*, WBC* white blood cell

^a^Comparison was made using Mann-Whitney U test at *P* < 0.05, and these values were described by median with inter-quartile range (25th and 75th percentile)

To examine the parameters associated with persistent LV systolic dysfunction, we designed univariate logistic regression analysis. Univariate analysis demonstrated that initial LV EF, WBC count, HDL, monocyte count, CRP, and MHR were associated with persistent LV systolic dysfunction (Table 2).

**Table 2:** Univariate logistic regression analyses for prediction of nonrecovery

Variable	OR	95% CI	*P* value
Age	1.077	0.918–1.265	0.362
Hypertension	1.380	0.320–5.957	0.666
Dyslipidemia	2.568	0.613–10.752	0.197
Family history of PPCM	5.793	0.655–51.235	0.114
Baseline LVEF	0.831	0.749–0.921	**< 0.001**
ACEI /ARB	1.007	0.273–3.711	0.992
B-blockers	1.043	0.307–3.541	0.946
Digoxin	1.440	0.475–4.372	0.519
Heart rate	1.005	0.969–1.042	0.789
Systolic blood pressure	0.990	0.952–1.029	0.604
Diastolic blood pressure	0.991	0.909–1.080	0.840
Body- mass index	0.952	0.854–1.060	0.371
HDL-C	0.938	0.899–0.979	**0.003**
CRP	1.247	1.061–1.465	**0.007**
WBC	1.407	1.077–1.839	**0.012**
Monocyte	1.976	1.309–2.983	**0.001**
Monocyte/HDL ratio	1.198	1.069–1.343	**0.002**
Monocyte/HDL ratio < 9.73	0.079	0.022–0.285	**< 0.001**

Bold data displays statisticially significant difference (*p* < 0.05)

*ACEI* angiotensin-convertingenzyme inhibitör*, ARB* angiotensin receptor blocker*, CI* confidence interval*, CRP* C reactive proein, *HDL-C* high density lipoprotein cholesterol*, LVEF* left ventricular ejection fraction, *OR* odds ratio, *PPCM* peripartum cardiomyophaty,*WBC* White blood cell

**Fig. 2:**
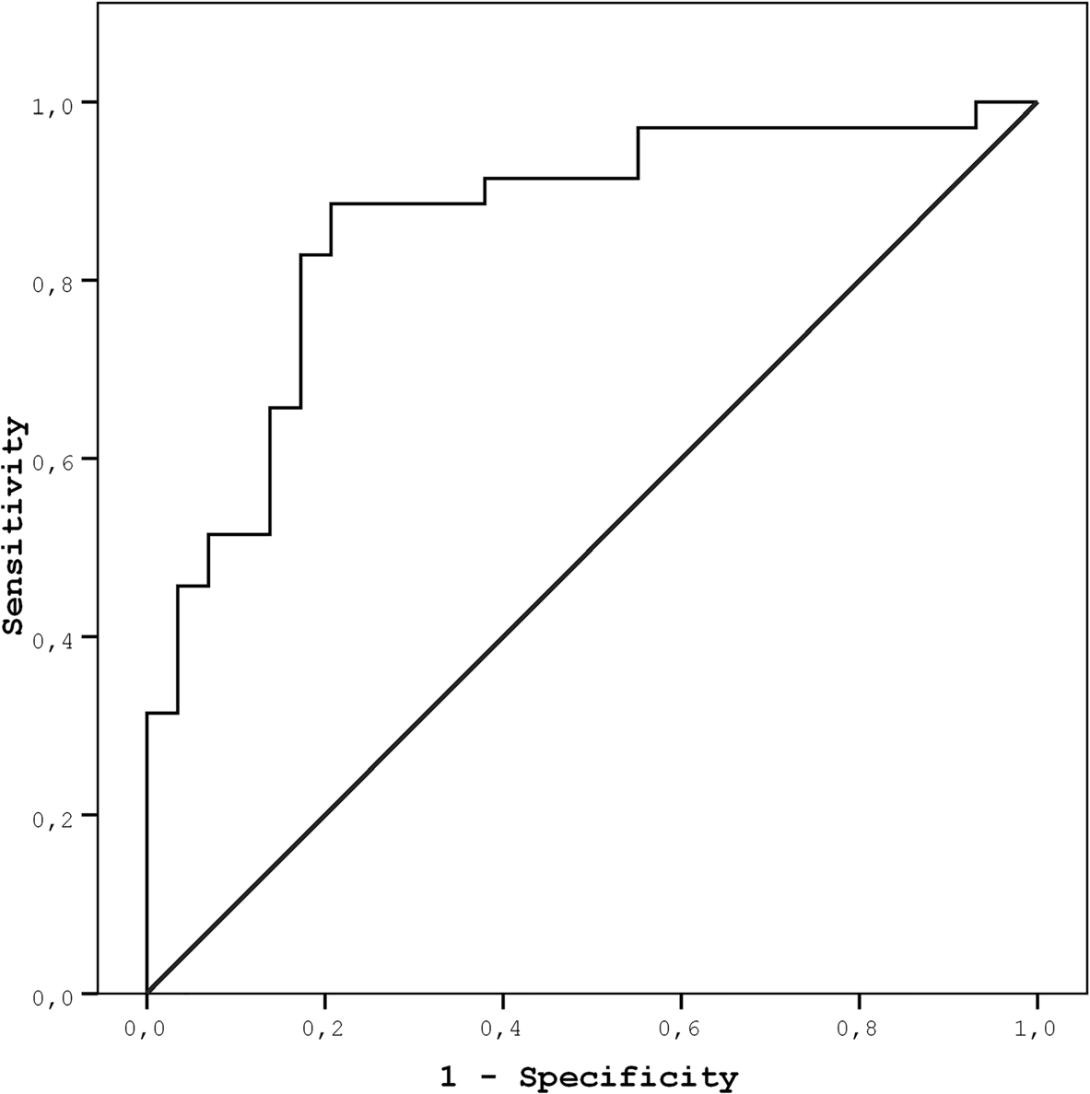
Receiver-operating characteristic curve of the Monocyte to-HDL-cholesterol ratio for predicting persistent left ventricular systolic dysfunction

The ROC curve analysis explored the discriminatory capability of admission MHR for the LV recovery. Area under the curve was 0.861 (95% CI: 0.768–0.954; *P* < 0.001). Using a cutoff level of 9.73, MHR predicted persistent LV systolic dysfunction with a sensitivity of 89% and specificity of 79% (Fig. 2).

## Discussion

In the present study, it was found that admission MHR values were significantly higher in the non-recovery group compared with the recovery group. Higher baseline CRP and WBC levels and lower baseline LV EF in addition to higher baseline MHR were significant predictors of LV recovery. To our knowledge, our study is the first in the literature investigating the possible relation between MHR and PPCM up till now.

The outcomes of PPCM differs widely. PPCM is a particular type of cardiomyopathy with the greatest possibility of myocardial recovery. It was shown that many patients with PPCM recover LV function partially or entirely, nonetheless failure to recover can be associated with significant adverse events and death [1, 11, 12]. Unfortunately, there are no accurate and exact predictors of whether or not myocardial recovery will occur. The attempts of clinical researchers to identify baseline predictors of poor outcomes in women with PPCM has culminated in the establishing of several predictors with moderate and inconsistent associations with prognosis.

Several studies have shown a correlation between a more depressed LV EF at initial diagnosis and a worse outcome in these patients [13, 14]. In addition, previous studies have reported a relation between an increased LVEDD, increased LVESD (left ventricular end-systolic diameter) on the initial echocardiogram, lower systolic blood pressure, higher resting heart rate and persistent LV dysfunction [15–17]. In our study, only lower baseline LV EF from echocardiographic findings was found a significant predictor of persistent LV dysfunction.

The exact pathophysiological mechanism that leads to PPCM is unknown, but increased oxidative stress and inflammation have been proposed in the pathogenesis of manifest cardiomyopathy. Recently, it was postulated that an oxidative stress– cathepsin D–16-kDa prolactin cascade is related to the pathophysiological mechanism of PPCM. During peri/postpartum period, enhanced oxidative stress that triggers the proteolytic cleavage of the prolactin into a potent anti-angiogenic, pro-apoptotic and proinflammatory 16-kDa prolactin fragment seems to play a central role in decreasing cardiomyocyte metabolism [18].

Inflammation can be measured using a variety of hematological and biochemical markers. In a recent study, Sarojini et al. found that the baseline IL-6, CRP, and TNF-alpha were relevant to the mortality in PPCM patients [19]. In another study, Gleicher et al. have demonstrated evidence of an inflammatory process characterized by cytokine imbalance associated with PPCM [20]. Sliwa et al. found that plasma marker of apoptosis (Fas/Apo-1) was relevant to the clinical course of this disease [21]. However, in these studies, the role of MHR, as an easily accessible new inflammation-based marker has not been assessed in predicting LV recovery. It is widely accepted that monocyte activation is strongly implicated in chronic inflammation and almost every aspect of cardiovascular diseases [22, 23]. Under certain stimuli, circulating monocytes transform into macrophages. Monocytes and monocyte-derived macrophages can trigger an inflammatory cascade involving the production of cytokines [24]. It has been suggested that such cytokines migrate to the myocardium and adhere to the endothelial wall. Therefore, infiltration of the myocardium eventually results in fibrosis and HF [25, 26].

On the other hand, high-density lipoprotein cholesterol (HDL) molecules counteract these pro-inflammatory and pro-oxidant effects of monocytes by impeding the migration -activation of monocytes and proliferation-differentiation of the progenitor cells of monocytes [27–29]. Thus, monocytes display pro-inflammatory and pro-oxidant effects, but HDL acts as a reversal factor during those processes. Hence, it is reasonable to combine these two parameters into a single ratio as oxidative stress and inflammation- based marker. Recently, MHR has emerged as a new and widely available cardiovascular prognostic marker. Its association of cardiovascular diseases (CVDs) has been examined in a few studies. The first study of Kanbay et al. reported that higher MHR has been associated with worse cardiovascular outcomes in patients with chronic kidney disease [30]. Another study suggested that, in patients with acute ST-segment elevation myocardial infarction (STEMI), the admission MHR values were independently correlated with in-hospital major adverse cardiovascular events (MACEs) and stent thrombosis as well as mortality [31]. In two different studies evaluating MHR in patients with stable or unstable angina pectoris who were undergone percutaneous coronary intervention with bare-metal stent implantation, investigators found a high level of admission MHR values were related with in-stent restenosis [32, 33]. Similarly, Kundi et al. [34] demonstrated that MHR was significantly higher in patients with coronary artery ectasia (CAE) and associated with the severity of CAE. Besides that, in a new study, MHR was found to be associated with CVD and the severity of obstructive sleep apnea syndrome (OSAS) [35].

All these findings show the importance of MHR in inflammation which has an essential role in the development of many cardiovascular events. In conjunction with the literature, our results may suggest that inflammation and oxidative stress have a vital role for PPCM pathophysiology and parameters which can directly or indirectly reflect inflammation can play a substantial role in pathophysiology and prognosis of PPCM. In our study, we found that average MHR values of the patients with persistent LV systolic dysfunction were prominently higher than the patients with LV recovery. One may hypothesize that MHR may predict recovery and make clinical decision making easy as to whether a woman with initially low EF may recover. This parameter may be used to establish patients at high risk for adverse outcomes and guiding selection for the type of therapy in patients with PPCM. From a clinical point of view, as a new predictor of inflammation and oxidative stress, special attention should be paid to MHR whenever evaluating a woman with PPCM at initial evaluation.

## Limitations

The most important limitation of the study was its retrospective cross-sectional design. Although a relatively large series of patients with PPCM were examined, the study population was small in size due to the lack of PPCM. Therefore, the small sample size may limit the power of a statistical test in revealing significant predictors. The data were collected from medical records of patients, and different physicians provided the echocardiographic examinationsphysicians. Monocyte and HDL counts were calculated automatically via venous blood samples. Also, prolactin and other other inflammation and oxidation parameters, such as IL-6 and TNF-alpha, were not assessed because they are not usually available in daily practice. Also, this study did not contain a control group. This limitation may lower the power of the study to determine the prediction of the cut-off value for MHR in this specific population. Finally, our study took place in one tertiary center, which may decrease the generalizability of our results.

## Conclusion

Our findings revealed that higher MHR levels were significantly associated with persistent LV systolic dysfunction in PPCM. These results suggest that higher MHR levels may represent a pro-oxidant and pro-inflammatory effect on the myocardium of these patients. As low-cost, simple, reproducible parameters of the CBC and lipid panel, the MHR can be widely used in clinical practice for prediction of LV recovery. However, our findings should be confirmed in prospective, randomized, large-scale studies involving other inflammatory biomarkers to explain the exact role of MHR in PPCM clearly.

## Abbreviations

CAD: Coronary artery disease
CAE: Coronary artery ectasia
COPD: Chronic obstructive pulmonary disease
CRP: C-reactive protein
CVD: Cardiovascular disease
DM: Diabetes mellitus
EF: Ejection fraction
HDL: High-density lipoprotein
HT: Hypertension
ICD: Intracardiac defibrillator
LV: Left ventricular
LVEDD: Left ventricular end-diastolic diameter
LVSD: Left ventricular systolic dysfunction
MHR: Monocyte count to high-density lipoprotein cholesterol ratio
OSAS: Obstructive sleep apnea syndrome
PPCM: Peripartum cardiomyopathy
STEMI: ST-segment elevation myocardial infarction
UA: Uric acid
